# Scale of employees’ initiative for proactive health: development and validity testing

**DOI:** 10.1186/s40359-025-03040-0

**Published:** 2025-07-01

**Authors:** Qianqian Huang, Feng Jiang, Huanzhong Liu, Lufa Zhang, Yi-Lang Tang

**Affiliations:** 1 School of International and Public Affairs, Shanghai Jiao Tong University, No.1954 Huashan Road, Xuhui District, Shanghai, China; 2 Institute of Healthy Yangtze River Delta, Shanghai Jiao Tong University, Shanghai, China; 3 Institute of Health Policy, Shanghai Jiao Tong University, Shanghai, China; 4 Department of Psychiatry, Chaohu Hospital of Anhui Medical University, Hefei, China; 5 Department of Psychiatry, School of Mental Health and Psychological Sciences, Anhui Medical University, Hefei, China; 6 Anhui Psychiatric Center, Hefei, China; 7 Department of Psychiatry and Behavioral Sciences, Emory University, Atlanta, GA USA; 8 Atlanta VA Medical Center, Decatur, GA USA

**Keywords:** Proactive health, Employees’ initiative, Factor analysis, Instrument development, China

## Abstract

**Background:**

Non-communicable diseases (NCDs) pose a significant public health challenge, with employees particularly vulnerable due to modifiable lifestyle risk factors. Proactive health underscores the importance of self-initiated behaviors in disease prevention and health promotion. However, existing measures primarily assess workplace initiative, overlooking its relevance to health. This study aims to develop and validate the Employees’ Initiative for Proactive Health (EIPH) scale to address this gap.

**Methods:**

The instrument development phase identified 11 preliminary items through literature review and expert consultation with five experts. The item discrimination index, item-total correlations, preliminary reliability, and exploratory factor analysis (EFA) were performed on a pilot sample of 204 participants, followed by confirmatory factor analysis (CFA) and concurrent validity on the main sample of 289 participants. The cutoff value was also assessed.

**Results:**

The EIPH scale was refined to five items, with eigenvalues greater than 1, accounting for 45.71% of the variance. Standardized factor loadings for the CFA indices ranged between 0.41 and 0.55. The correlation coefficient for concurrent validity was 0.569 (*p* < 0.001), and Cronbach’s α coefficient for reliability was 0.58. The cutoff value was 18.5 points.

**Conclusions:**

The scale demonstrated reasonable and reliable measurement of EIPH among Chinese participants.

## Introduction

Non-communicable diseases (NCDs) are primary contributors to global health challenges and are responsible for 70% of deaths worldwide [1]. In China, NCDs account for 86.6% of all deaths [2]. Behavioral risk factors, including unhealthy diet, smoking, and physical inactivity, which are particularly pronounced among employees, are modifiable contributors to the development of NCDs [3]. Effective referral pathways for preventing and managing NCDs are essential in the context of escalating health demands and resource constraints, which are exacerbated by rapid socio-economic development and transformative technological advancements.

In this involving landscape, “proactive health” has become a transformative service model in the public health domain, aiming at improving human health [4]. This approach indicates a paradigm shift from reactive to proactive health management strategies, encompassing all social activities centered around health. It emphasizes controlling health risk factors at their source and actively responding to population health crises. At its core, proactive health embodies a comprehensive and participatory approach to health and wellness. The essence is the proactivity of people, where the public takes the initiative in disease prevention [5].

Employees’ initiative, or proactivity, defined as employees’ self-starting and proactive action towards goal achievement [6], which is widely applied in the field of work engagement, is also essential in health maintenance and promotion. It significantly influences employees’ health behaviors, guiding how employees engage in preventive measures, seek information, and adopt practices to promote health [7]. At the same time, it can also be influenced by geographic location, individual burden, disparities in healthcare provision, and socio-economic inequalities [8, 9]. This concept encapsulates the actions taken by employees, communities, and healthcare providers to prevent diseases, promote health, and respond to health risks effectively. By fostering employees’ initiative, individuals are empowered to take charge of their health, make informed decisions, and engage in health-promoting behaviors.

Prioritizing the significance of employees’ health is crucial, for it not only fosters employees’ productivity and commitment but also improves the organization’s outcome and business reputation [10]. In this case, assessing employees’ initiative within the realm of health, particularly proactive health, has emerged as a pivotal factor in contemporary public health research and practice. The importance of quantifying personal initiative lies in its potential to provide insights into the underlying mechanisms driving proactive health behaviors, which, in turn, may guide the development of targeted interventions to improve employees’ health outcomes. Grossman’s Health Human Capital Theory underscores health as a fundamental component of human capital, emphasizing that a workforce with high levels of health-related human capital is crucial for ensuring a stable, sustainable, and high-quality labor supply [11, 12]. This is especially pertinent in developing countries, where deficiencies in healthcare infrastructure and medical security systems render labor market participation and economic returns more vulnerable to the influence of health human capital. Furthermore, research suggests that employees with higher personal initiative levels are more likely to pursue activities that promote physical and mental health, exhibit resilience in the face of health challenges, and take an active role in managing their healthcare management [13, 14]. Therefore, assessing employees’ health initiative contributes to a deeper understanding of health behavior and the development of more effective health promotion programs and policies. By focusing on enhancing employees’ initiative, public health efforts can foster a more proactive and self-reliant, empowered population. Despite the importance of personal initiative in health as mentioned above, there is a gap in its assessment of proactive health. While Frese et al. developed a questionnaire-based scale of personal initiative in Germany [15], it focused on the employees’ initiative for work. Similarly, Bateman et al. merely proposed measures of individual proactive behavior among American undergraduates [16]. Furthermore, scarce comparable scales have been developed or validated in developing countries. Therefore, in this study, we aimed to develop a self-report scale to assess employees’ initiative for proactive health (EIPH) in China.

## Design

The scale of EIPH was developed and validated across two distinct phases: the instrument-development phase and the instrument-validating phase. These phases align with the scale development guidelines established by DeVellis and Thorpe [17].

## Phase 1: Instrument development

### Item composition

To identify the items of the instrument, two researchers independently reviewed both English and Chinese literature on the attributes and characteristics of EIPH. The literature was sourced from five databases: PubMed, Web of Science Core Collection, Google Scholar, Scopus, and China National Knowledge Infrastructure (CNKI), covering publications from January 1997 to March 2024. The first four databases were used to retrieve English-language literature, while CNKI was used to search Chinese-language sources. Qualitative content analysis techniques were employed only in Chinese for data analysis [18].

The literature review identified four key attributes of EIPH: health responsibility, healthy lifestyle, health information or guidance, and health target. Based on these attributes, 15 initial items were created.

### Content validity verification

An expert content validity assessment was conducted for the 15 initial items. The panel of experts consisted of five experts (professors) specializing in public health, public management, psychology/psychiatry, and sports sciences. Each expert rated the suitability of the initial items for measuring personal initiative for proactive health using a 5-point scale: 1 (strongly disagree), 2 (disagree), 3 (neutral), 4 (agree), and 5 (strongly agree).

The item-level content validity index (ICVI) was calculated, aiming for a minimum score of 0.78, as recommended by Lynn (1986) [19]. In this process, 4 items were eliminated based on the feedback from the expert panel, leaving 11 items to proceed to the next phase (See Table 1).

**Table 1:** 11 preliminary items

Item	Content
1	I agree with the following statement: “Individuals are the first person responsible for their own health.”
2	I take the initiative to undergo regular physical examinations.
3	I take the initiative to learn information that can maintain and improve my health.
4	I will take the initiative to change unhealthy eating patterns (such as high-oil and high-fat diets, etc.)
5	I will take the initiative to change sleeping patterns that are not conducive to health (such as going to bed late, getting up late, etc.)
6	I will take the initiative to participate in physical exercise to improve my health (such as walking, playing ball, yoga, etc.)
7	I will do some things that can bring me short-term happiness but may be harmful to my body (such as smoking, etc.)
8	I constantly improve my lifestyle to keep myself in better health.
9	Even when I am very busy, I still undergo regular physical exercise.
10	I actively seek health guidance from others when needed.
11	It is difficult for me to make a plan for my future health.

## Phase 2: Scale validation

### Participants

The predefined sample size for this survey was determined to be more than 200, considering a dropout rate based on a previous study [17]. This number aligns with recommended guidelines suggesting 5–10 participants per item [20] and is adequate for factor analyses involving fewer than 40 items [21]. The inclusion criteria for the participants were: (1) employees aged between 18 and 60 years old; (2) Chinese nationality and permanent residents of China; (3) ability to complete the online questionnaire; and (4) understanding of each questionnaire item. The exclusion criteria were inability to communicate or difficulties filling out the questionnaire. We conducted both a pilot study for exploratory factor analysis (EFA) and a main study for confirmatory factor analysis (CFA).

### Data collection

The pilot study was conducted from February 3 to 10, 2024, and the main study from March 5 to 15, using a snowball sampling method. Survey announcements and website links were posted on workplace bulletin boards, encouraging participants to complete and share the survey. Questionnaires were distributed and collected through Wenjuanxing (www.wjx.cn). Participants were recruited from Shanghai city for the pilot study and from across mainland China for the main study. To prevent duplicate responses, each Internet Protocol (IP) address was limited to one survey submission. Participation was voluntary and anonymous, with all respondents providing their informed consent before starting the survey.

**Fig. 1:**
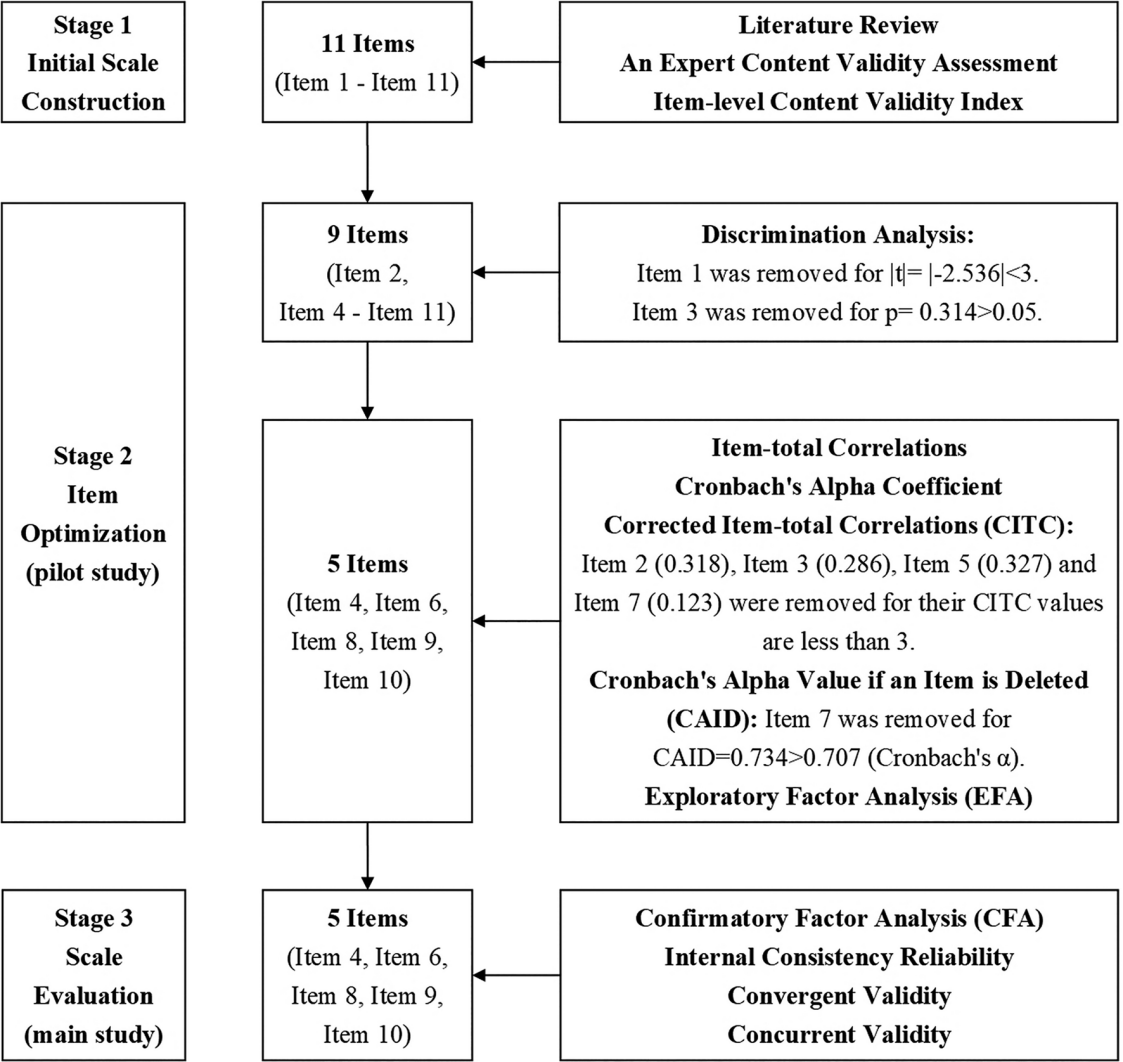
The procedure of the EIPH scale development

Basic sociodemographic information was collected based on the literature review [22]. A five-point Likert scale was used to test whether participants agreed with each item. Figure 1 illustrates the development process of the EIPH scale.

The final analyses included data from 204 participants in the pilot study and 289 in the main study after excluding incomplete questionnaires (*N* = 20 for the pilot and 54 for the main studies, respectively).

### Statistical analysis

In the pilot study, we used SPSS 19.0 (IBM, Armonk, NY, USA) to compute the item discrimination index, item-total correlations, preliminary reliability, and EFA. In the main study, we used SPSS 19.0 to calculate internal consistency reliability and IBM SPSS AMOS 23.0 (IBM, NY, USA) for CFA and concurrent validity. Furthermore, the Mplus Version 8.1 (Muthén and Muthén, 1998–2017) and the “pROC” package in R 4.3.3 (R Foundation for Statistical Computing, Vienna, Austria) were applied to conduct latent profile analysis (LPA) and receiver operating characteristic (ROC) analysis, respectively.

#### Item evaluation

We used classical test theory methods, including item discrimination analysis, item-total correlations, preliminary reliable test, and EFA in SPSS 19.0 to quantitatively evaluate and screen the scale items. The screening criteria were as follows: (1) discrimination indices: The item discrimination index measures how well an item on a rating scale differentiates between respondents with high and low scores on the overall scale [23]. Participants were ranked based on their total scores, with the top 27% forming Group 1 and the bottom 27% forming Group 2. A *t*-test was conducted to compare the scores of each item between the two groups. Referring to the existing research, items with no significant difference in *t*-test scores (*p* > 0.05) and critical ratio < 3 were considered for elimination [17, 23–25]. (2) Item-total correlations: In order to examine the relationship between each item and the total score of the scale, the item-total correlation is preferred. Items with very low correlations (< 0.40) are generally less suitable and are often eliminated from the tentative scale [17, 26]. (3) preliminary reliable test: reliability refers to the consistency and stability of measurement results. A higher reliability coefficient indicates more consistent and stable results. Cronbach’s α coefficient is generally used to measure the reliability of a scale. At the same time, items are also assessed based on the corrected item-total correlations (CITC) and the Cronbach’s α value if an item is deleted (CAID). Previous literature implies that indicators with a CITC coefficient less than 0.4 and a CAID coefficient greater than the overall Cronbach’s α of the scale should be removed [24, 27–29]. (4) EFA: if the Kaiser-Meyer-Olkin (KMO) value exceeds 0.7 and Bartlett’s sphericity test is statistically significant (*P* < 0.05), factor analysis is considered feasible [30]. Items with factor loadings < 0.40 on their common factors were deleted [31].

#### Construct validity and convergent validity

CFA was conducted to assess the goodness of fit between the proposed model and the collected data [32, 33]. Based on the factor solution obtained from the EFA, we performed CFA in the main study (*n* = 289) to test the scale’s construct validity. The model fit was evaluated using the χ^2^ over degrees of freedom (χ^2^/df), root mean square residual (RMR), goodness-of-fit index (GFI), iterative fit index (IFI) and root mean square error of approximation (RMSEA). A good fit is indicated when the GFI and IFI are both greater than 0.9, and the RMR and χ^2^/df are less than 0.08 and 5, respectively [34–36]. As to RMSEA, although it is widely recognized that a value below 0.05 indicates a close fit [37], some senior researchers have suggested that a model should be considered to exhibit poor fit and be deemed unacceptable only when the RMSEA exceeds 0.10 [38–40]. Especially, models with small degrees of freedom and limited sample sizes tend to produce inflated RMSEA values [41]. Given the short length of the scale developed in our study, we adopted 0.10 as the threshold for RMSEA. What’s more, standardized factor loadings were expected to be above 0.4 [42].

We also assessed Cronbach’s α coefficient for internal reliability, and composite reliability (CR) and average variance extracted (AVE) for convergent validity. Although acceptable thresholds are typically CR ≥ 0.70 and AVE ≥ 0.50 [43, 44], convergent validity is still regarded as adequate if the AVE value is less than 0.50 but the CR value is greater than 0.60 [45–49].

#### Concurrent validity

To examine concurrent validity, the Personal Initiative Scale (PIS) for employees was involved as the benchmark. This scale encompasses dimensions such as work initiative, action orientation, problem-focused coping, and executing plans in their roles [15]. Focusing on individuals’ work initiative, the PIS has 7 items with a five-point Likert scale. And the Chinese version of PIS has moderate internal reliability [50–53]. The Pearson correlation coefficient (r) was used to test whether there was a significant correlation between the EIPH scale and PIS. The criterion for acceptable correlation is|r| ≥ 0.40 [54].

#### Scale cutoff point validation

To find out the cutoff point of the EIPH scale, the LPA has been proposed to determine the classification of individuals and, thus, to derive essential sensitivity and specificity in the absence of an accurate reference standard. Using the 5 items of EIPH as indicators, LPA was performed through robust maximum likelihood estimation to identify distinct patterns of proactive health among employees. A series of LPA models were fitted and compared to determine the best fitting model. As recommended by previous studies, the optimal model was selected based on several fit indices, including the Akaike information criterion (AIC), Bayesian information criterion (BIC), sample-size adjusted Bayesian information criterion (aBIC), bootstrap likelihood ratio test (BLRT), Lo-Mendell-Rubin test (LMRT), and entropy [55, 56]. Generally, lower AIC, BIC, and aBIC values indicate a relatively better fit. Entropy, a standardized measure of how individuals are assigned to correct latent groups, was regarded as indicative of classification accuracy where an entropy value of 0.80 or higher represented the adequate quality of classification [56, 57]. The LMRT and BLRT were employed to test the discrepancy between two models (i.e., k-class vs. k-1-class), and significant P-values (*p* < 0.05) provided support that the k-class was preferable [56–58]. Taken together, the number of latent profiles was determined by a combination of the above fit criteria, rather than a single one.

After that, the ROC analysis was performed to determine the optimal cutoff value for the EIPH scale [59]. The performance of the classifiers was evaluated by the area under the ROC curve (AUC), sensitivity, specificity, and Youden’s index value (sensitivity + specificity-1). Among those measures, the AUC was used as a measure of diagnostic accuracy, and it indicates perfect diagnostic power when an AUC value is close to 1. The optimal cutoff point was identified based on the maximum Youden’s index [60]. All analyses in the study were completed with Mplus version 8.1 and R version 4.3.3.

### Ethical considerations

The ethical approval was granted by the Ethics Committee of Shanghai Jiao Tong University (IRB No. H20230308I). Data collection began after obtaining informed consent from participants who understood and voluntarily agreed to the study objectives.

## Results

### Participant characteristics

Demographic data, including gender, age, and educational level, was presented in Table 2. The gender distribution in both surveys was approximately equal, with a nearly 50% ratio. The largest proportion of participants was 30–39 years old (56.4% and 63.3% in the pilot and main studies respectively). More than 85% of participants completed a college education or above.

**Table 2:** General characteristics of the participants

Characteristics	Categories	Pilot study (204)		Main study (289)	
		N	%	N	%
Gender	Male	105	51.5	115	39.8
	Female	99	48.5	174	60.2
Age(years)	20–29	40	19.6	74	25.6
	30–39	115	56.4	183	63.3
	40–49	43	21.1	29	10.0
	50–60	6	2.9	3	1.0
Education level	Below undergraduate	24	11.8	41	14.2
	Undergraduate	156	76.4	229	79.2
	Graduate	24	11.8	19	6.6
Region	East China	204	100.0	193	66.8
	Center China	-	-	43	14.9
	West China			39	13.5
	Northeast	-	-	14	4.8

### Item evaluation results

#### Discrimination indices and item-total correlations

For discrimination indices, item 1 (t=-2.536, *p* = 0.013) and item 11 (t = 1.012, *p* = 0.314) were removed, leaving 9 items for subsequent analysis. After that, we calculated item-total correlations and found that the correlation coefficients between each item and the total score ranged from 0.422 to 0.709 (*p* < 0.01), supporting the 9 items to be retained for further analysis.

#### Preliminary reliability test

In this study, a reliability test was conducted on 9 items, yielding an overall Cronbach’s α of 0.707. However, four items were eliminated due to low CITC: Item 2 (0.318), Item 3 (0.286), Item 5 (0.327), and Item 7 (0.123). Additionally, the CAID for Item 7 was 0.734, greater than the overall Cronbach’s α (0.707). Therefore, items 2, 3, 5, and 7 were removed. The remaining 5 items in the scale demonstrated acceptable CITC, ranging from 0.411 to 0.546 (See Table 3).

**Table 3:** CITC, CAID, and Cronbach’α of each item

Item	CITC	CAID	Cronbach’α
Item-2	**0.318**	0.663	0.707
Item-3	**0.286**	0.668	
Item-4	0.411	0.644	
Item-5	**0.327**	0.661	
Item-6	0.447	0.638	
Item-7	**0.123**	**0.734**	
Item-8	0.491	0.631	
Item-9	0.546	0.607	
Item-10	0.432	0.640	

#### Exploratory factor analysis

EFA was adopted to identify the latent structure of each item in the scale, and several established cutoffs were used to evaluate the outcome [61]. For the pilot study, the KMO measure of sampling adequacy was 0.708, and Bartlett’s test of sphericity was statistically significant (χ^2^ = 172.622, *p* < 0.01), supporting the factorability of the data. The factor loading for items ranged from 0.605 to 0.790, and the commonalities ranged from 0.367 to 0.625. Both values exceeded the recommended thresholds of 0.45 and 0.2, respectively [17, 24]. Therefore, all 5 items were retained. Table 4 presents the items with their loadings in each factor, and the cumulative variance explained was 45.708%.

**Table 4:** Rotated factor loadings for the scale

Factor	Item	factor loading	extraction of communalities	cumulative interpretation rate
EIPH	Item-4 (I will take the initiative to change unhealthy eating patterns.)	0.605	0.367	45.708%
	Item-6 (I will take the initiative to participate in physical exercise to improve my health.)	0.663	0.440	
	Item-8 (I constantly improve my lifestyle to keep myself in better health.)	0.637	0.405	
	Item-9 (Even when I am very busy, I still undergo regular physical exercise.)	0.790	0.625	
	Item-10 (I actively seek health guidance from others when needed.)	0.670	0.449	

### Confirmatory factor analysis results

**Fig. 2:**
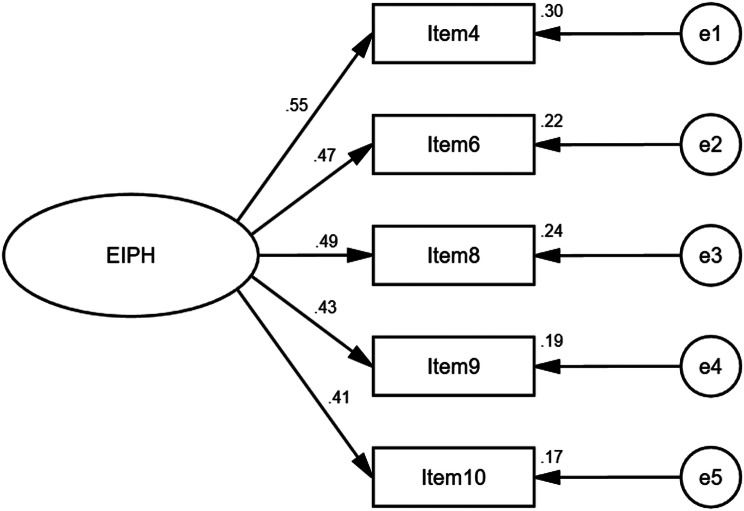
Structural model of the EIPH scale

CFA was conducted in the main study sample (*n* = 289), and our analysis generally confirmed the single-factor structure extracted during the EFA phase. Figure 2 shows the structural model with individual items and standardized coefficients of each path.

The CFA showed an overall satisfactory model-data fit for the EIPH scale, as indicated by the following goodness-of-fit indicators: RMR = 0.034, RMSEA = 0.091, GFI = 0.977, and IFI = 0.901, respectively. In addition, the χ^2^/df value was 3.384, indicating a good fit (< 5) [17].

The Cronbach’s α coefficient for all items was 0.58, indicating a satisfactory level of internal consistency reliability for the scale [17, 62]. To further evaluate the convergent validity of the EIPH scale, we calculated composite reliability (CR = 0.6013) and average variance extracted (AVE = 0.236). Even if AVE is less than 0.5, convergence validity is considered sufficient if CR exceeds 0.6. Therefore, the convergent validity of this measure was considered acceptable. The final scale is presented in Table 4.

### Concurrent validity results

We assessed concurrent validity by examining the correlation between the final version of EIPH (Items 4, 6, 8, 9, 10) and the PIS. The Pearson correlation coefficient was 0.569 (*p* < 0.001), showing a significant positive correlation between these scales.

### Scale cutoff point validation results

LPA was conducted with one-to-four-class solutions, and the fit indices for the four models were presented in Table 5. The AIC, BIC, and aBIC continuously decreased along increase in the number of latent profiles. And the value of entropy in each model was above 0.80, indicating that all models could provide high classification accuracy. However, the p-values of the Lo-Mendell-Rubin test (pLMRT) were all above 0.05 except for the 2-profile model, which implies that the models with 3 profiles or above don’t fit better than that with 2 profiles. What’s more, the p-value of the bootstrap likelihood ratio test (pBLRT) in the 2-profile model was less than 0.05, and the relative frequency of the smallest class exceeded 5%, both meeting the corresponding thresholds. Taken together, the 2-profile model was selected as the optimal solution [63]. Based on the average scores of each item, participants were categorized into the “Low Personal Health Initiative” group (13.1%) and the “High Personal Health Initiative” group (86.9%).

**Table 5:** Latent profile analysis results

Profile	AIC	BIC	aBIC	Entropy	pLMRT	pBLRT	Probability of classes (%)
1-profile	3504.853	3541.518	3509.806	-	-	-	-
2-profile	3377.564	3436.227	3385.489	0.845	0.016	0.000	13.1/86.9
3-profile	3012.170	3092.831	3023.066	1.000	0.483	0.000	18.3/49.5/32.2
4-profile	2989.451	3092.111	3003.318	0.997	0.114	0.000	1.0/48.4 /32.2/18.3

**Fig. 3:**
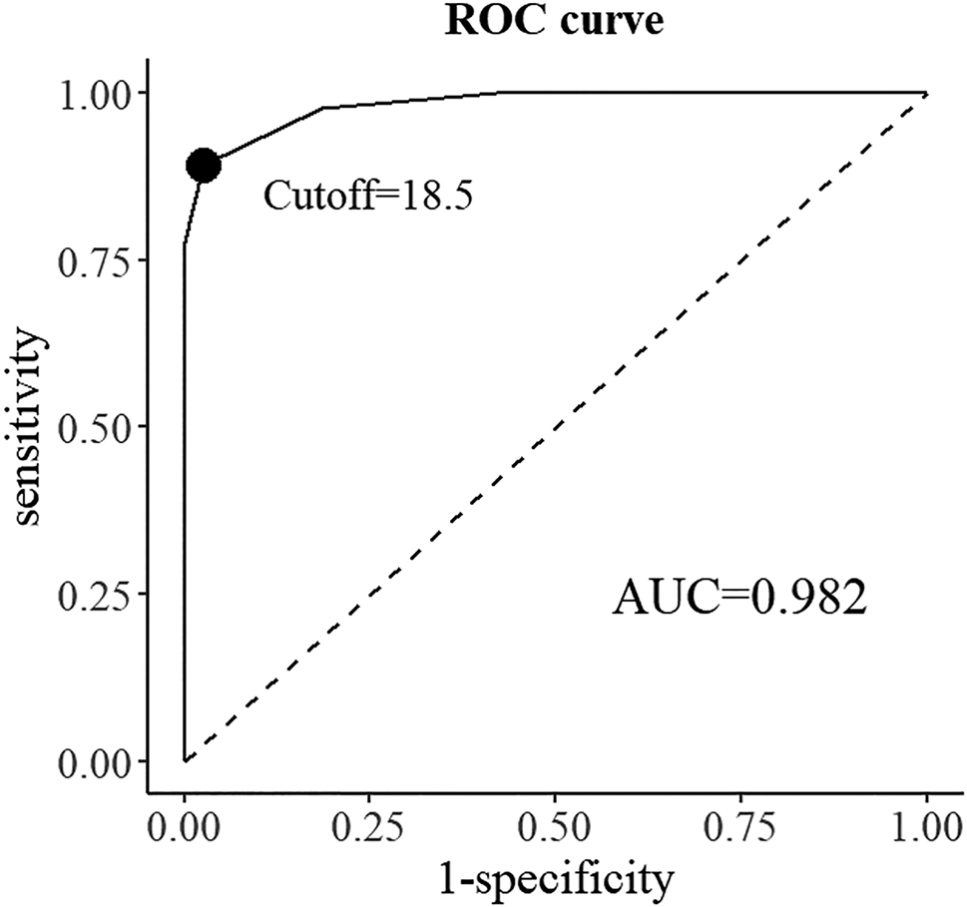
The ROC curve of the proactive health

Cutoff values were determined through the ROC curve analysis (see Fig. 3). Based on the LPA results, participants classified in the “Low Personal Health Initiative” group were considered “non-cases,” while those in the other group were designated as “cases”. According to the maximum Youden index (Youden’s index = 0.866), the optimal cutoff was determined to be 18.5, with sensitivity and specificity values of 0.892 and 0.974, respectively. The AUC was 98.2% (*p* < 0.001, 95% CI: 0.969–0.996), demonstrating an excellent predictive capacity for EIPH.

## Discussion

This study represents a pioneering effort to focus on the EIPH, filling a significant gap in current research. We developed a concise five-item EIPH scale capturing initiative in eating patterns, physical exercise, continuous improvement, health information acquisition, and barriers overcoming.

The concept of employees’ initiative refers to a behavioral pattern where an individual actively and independently engages in work activities, exceeding formal role expectations. This concept is defined by several key attributes: (1) alignment with the organizational goals, (2) a long-term strategic approach, (3) a focus on actionable objectives, (4) perseverance despite challenges and obstacles, and (5) a proactive and initiating attitude [6]. In the context of proactive health, personal initiative translates to employees actively pursuing health promotion and overcoming barriers [5]. Given that adherence significantly influences life expectancy and reduces major NCD risks [64], fostering personal initiative in proactive health is crucial.

Two items in the scale emphasized the pivotal role of physical exercise in health maintenance and disease prevention. Physical activity is universally recognized for its extensive health benefits, which include reducing the risk of heart disease, diabetes, and cancer [65]. It also promotes mental health by reducing depression and anxiety [66]. Regular physical activity exemplifies personal initiative in proactive health by proactively maintaining or enhancing one’s health status before the emergence of illness.

The item addressing overcoming difficulties encapsulates resilience in maintaining health initiatives despite challenges. Resilience is critical in proactive health, where consistent, long-term efforts often face immediate gratifications or obstacles [67]. The assessment of resilience can enhance interventions targeting individuals in high-stress environments or those with limited access to health resources.

Introducing the EIPH emphasizes the shift from traditional health metrics focusing on disease prevalence and treatment outcomes to a holistic approach encompassing proactive health behaviors and initiatives. Although existing literature addresses organizational practices and policies related to workplace health promotion, it primarily emphasizes factors at the organizational level [68], such as extended operational history, medium-to-large enterprise size, and allocated budgets for health promotion, to enhance employees’ health and subsequently improve organizational performance without paying insufficient attention to individual-level considerations. Our research focuses on stimulating employees’ motivation and proactive engagement with their own health, thereby enriching existing studies in this area.

The EIPH scale holds important implications for public health policy and human resource practice. First, it highlights the need for health promotion programs to incorporate strategies that encourage and enable individuals to take proactive steps toward their health. Second, the development of the EIPH scale offers a valuable tool for researchers, clinicians, and policymakers to assess the effectiveness of health promotion interventions. Third, given the high incidence of overwork-related fatalities among young people, personal initiatives for proactive health can help employees perform better at work.

To the best of our knowledge, this is the first study to develop and validate a scale assessing EIPH in China, though some limitations should be acknowledged. Firstly, the generalizability of our findings to broader populations was limited, as our participants were predominantly employees aged between 18 and 60 years old, thus excluding individuals over 60 and consequently overlooking the aging workforce in the context of global population aging. Moreover, our study did not adequately consider vulnerable groups facing challenges in accessing health services [8, 69, 70]. Secondly, caution should also be exercised regarding the scale’s applicability in different cultural contexts because our investigation was exclusively conducted in China. Thirdly, our research utilized the snowball sampling method, which carries the risk of overrepresenting certain groups due to its reliance on social networks. Specifically, given the current employment context characterized by a generally higher education level, participants mainly consisted of highly educated individuals, potentially leading to an overestimation of initiative scores for proactive health among Chinese employees. Fourthly, although the CFA supported the structural validity of the scale, the internal consistency reliability, as indicated by the alpha coefficient, was relatively low, likely due to the brief scale length. This limitation may affect the credibility of the 5-item EIPH scale for its lower level of inadequate precision, accuracy, and stability. Finally, the optimal cutoff value for the EIPH was identified using a sophisticated analytic method rather than formal and objective clinical indicators or external scales, which may diminish the persuasiveness of our results.

Considering these limitations, future research could address the following aspects to strengthen the validity and applicability of the EIPH scale. First, it is essential to expand the targeted population of the EIPH scale. For example, additional assessments such as standardized measures of work ability or physical fitness may be incorporated, especially for older workers [71]. Moreover, integrating well-established or adapted measures related to healthcare access would provide valuable comparative insights [8]. Furthermore, future research should validate the EIPH scale across diverse cultural contexts to examine its cross-cultural applicability and generalizability. Incorporating participants with varying educational levels and employing more representative sampling strategies would also further strengthen the scale’s representativeness. In parallel, subsequent studies could replicate reliability analyses using larger and more diverse samples to confirm and enhance the psychometric robustness of the scale. Lastly, future studies are encouraged to validate the identified optimal cutoff point against gold-standard objective indicators.

## Conclusion

The EIPH scale developed in this study is a valid, reliable instrument assessing the initiative characteristics of participation in lifestyle management activities. Our findings will generate scientific evidence for further research to explore strategies and develop health promotion and disease prevention interventions for the entire population. The EIPH scale can be a valuable tool for researchers and practitioners seeking to understand and promote active engagement in healthy lifestyle behaviors. It can inform the development of more effective health promotion interventions. Future studies should further validate and refine the EIPH in different populations, exploring its utility in designing and evaluating programs that empower individuals to take an active role in managing their health and wellness.

## Data Availability

The datasets analysed during the current study are not publicly available due to ethical protection but are available from the corresponding author on reasonable request.

## Abbreviations

NCD: Non-communicable Disease
EIPH: Employees’ Initiative for Proactive Health
EFA: Exploratory Factor Analysis
CFA: Confirmatory Factor Analysis
CNKI: China National Knowledge Infrastructure
ICVI: Item-level Content Validity Index
IP: Internet Protocol
CITC: Corrected Item-Total Correlations
CAID: Cronbach’s Alpha Value if an Item is Deleted
LPA: Latent Profile Analysis
ROC: Receiver Operating Characteristic
KMO: Kaiser-Meyer-Olkin
RMR: Root Mean Square Residual
GFI: Goodness-of-Fit Index
IFI: Iterative Fit Index
RMSEA: Root Mean Square Error of Approximation
CR: Composite Reliability
AVE: Average Variance Extracted
PIS: Personal Initiative Scale
AIC: Akaike Information Criterion
BIC: Bayesian Information Criterion
aBIC: adjusted Bayesian Information Criterion
LMRT: Lo-Mendell-Rubin Test
BLRT: Bootstrap Likelihood Ratio Test
AUC: Area Under the Curve
CI: Confidence Interval
pLMRT: The P-value of the Lo-Mendell-Rubin Test
pBLRT: The P-value of the Bootstrap Likelihood Ratio Test
